# Trends in cost-related forgone care among older adults in Switzerland: a repeated cross-sectional study

**DOI:** 10.1093/eurpub/ckag010

**Published:** 2026-02-11

**Authors:** Mathieu Jendly, Stéphane Cullati, Cornelia Wagner, Axelle Braggion, Valérie Santschi, Arnaud Chiolero

**Affiliations:** Population Health Laboratory (#PopHealthLab), University of Fribourg, Fribourg, Switzerland; Population Health Laboratory (#PopHealthLab), University of Fribourg, Fribourg, Switzerland; Swiss Centre of Expertise in Life Course Research, Geneva & Lausanne, Switzerland; Swiss School of Public Health (SSPH+), Zurich, Switzerland; Population Health Laboratory (#PopHealthLab), University of Fribourg, Fribourg, Switzerland; Swiss Centre of Expertise in Life Course Research, Geneva & Lausanne, Switzerland; Swiss School of Public Health (SSPH+), Zurich, Switzerland; Population Health Laboratory (#PopHealthLab), University of Fribourg, Fribourg, Switzerland; Swiss School of Public Health (SSPH+), Zurich, Switzerland; La Source, School of Nursing Sciences, HES-SO University of Applied Sciences and Arts Western Switzerland, Lausanne, Switzerland; Population Health Laboratory (#PopHealthLab), University of Fribourg, Fribourg, Switzerland; Swiss School of Public Health (SSPH+), Zurich, Switzerland; School of Population and Global Health, McGill University, Montreal, QC, Canada; Institute of Primary Health Care (BIHAM), University of Bern, Bern, Switzerland

## Abstract

**Background:** Ensuring equitable healthcare provision is key in ageing societies, yet it may be hindered by financial barriers. We assessed trends and socioeconomic disparities in cost-related forgone medical care among Swiss adults aged 65 years and older between 2017 and 2024. **Methods:** We used data from the 2017, 2021, and 2024 waves of the ‘International Health Policy Survey’, a population-based study of randomly sampled adults aged 65 or older (*n* = 2570, 1888, and 1948, respectively). Participants reported whether they had forgone medical prescriptions, consultations, medical tests, treatments or follow-up consultations, and dental visits due to cost. Weighted prevalence estimates were computed for services covered by the basic insurance and for dental care. Disparities by education and income were assessed using stratified analyses and the index of disparity. **Results:** Participants’ characteristics were stable across all waves (mean age 75; 54% women). In 2024, 20% reported forgoing at least one service due to cost (13% forgoing dental care, 13% insurance-covered services). Forgone care was similar in 2017 (21%) and lower in 2021 (16%). Forgone care was more frequent among men and participants aged 65–79 years. The index of disparity showed widening income-related disparities over time, while disparities by education remained stable. Dental care consistently showed the largest disparities. **Conclusion:** Despite Switzerland’s compulsory health insurance, one in five older adults still forgo care for financial reasons. Rates of forgone care remained stable, but income disparities have widened since 2017.

## Introduction

Ensuring equitable healthcare provision is a key priority in ageing societies. Multiple barriers may limit access to healthcare in older adults, including financial constraints, limited insurance coverage, or geographic distance from health services [1]. These challenges are particularly important as multimorbidity and functional limitations are common among older adults and often require regular access to healthcare providers. Like other high-income countries, the Swiss population is ageing [2, 3], and the number of people aged 65 years and older is growing rapidly [4]. As life expectancy increases, healthcare needs are becoming more complex and frequent due to the growing burden of chronic diseases [5].

One important challenge related to equitable healthcare provision is forgone care, which is one dimension of not-chosen unmet need [6]. Unmet need is a broader concept, linked to limited healthcare access, resulting from various barriers, including financial constraints, long waiting times, lack of time, or fears [7]. Forgone care can include services covered by many basic insurance plans, such as preventive care or regular doctor appointments, which may require out-of-pocket payments, e.g. due to deductibles or copayments. It also encompasses non-reimbursed services, such as dental care, which is often excluded from basic insurance coverage. Forgone care due to cost disproportionately affects individuals with lower income, or indirectly lower education, contributing to persistent inequalities in health [8]. It is associated with poorer control of diabetes, arterial hypertension, or dyslipidaemia [9]. Forgone care is a persistent challenge, including in high-income countries like Switzerland, where access should be guaranteed by the national compulsory health insurance. Nevertheless, cost-sharing is among the highest in the OECD, which have been shown to be associated with increased forgone care [10]. In Switzerland, a previous study has shown that forgone care affected about 10% of the patient population in primary care practices [11].

Measuring disparities in health service uses is central to monitor equity and guide policy interventions but comes with many challenges. There is no single optimal indicator for socioeconomic position [12, 13] and multiple tools exist to assess health disparities. The index of disparity, as described by Pearcy and Keppel (2002) offers a simple relative measure that captures the average deviation of group-specific rates from the overall rate [14]. It is useful for comparing different indicators and multiple population subgroups, and tracking disparities, for instance in forgone care. In Switzerland, the disparities between older individuals of lower and higher socio-economic position in cost-related forgone care remain uncertain. Therefore, we assessed trends and socioeconomic disparities in cost-related forgone medical care among Swiss adults aged 65 years and older using data from the 2017, 2021, and 2024 International Health Policy (IHP) surveys conducted by the Commonwealth Fund.

## Methods

### Study design

We conducted a secondary analysis of the 2017, 2021, and 2024 waves of the International Health Policy (IHP) Surveys of the Commonwealth Fund (CWF) [15]. The CWF is a non-profit foundation in the United States that has been conducting IHP surveys since 1998 to compare the health system performances in several OECD (Organization for Economic Co-operation and Development) countries. One at a time, three target groups are surveyed every three years: the resident population aged 18 years and over, the resident population aged 65 years and over, and primary care physicians.

In 2017, 2021, and 2024, the CWF surveyed the population aged 65 and over in Switzerland and other OECD countries. A similar IHP survey was carried out in 2014. However, we did not include this wave as the 2014 survey included participants aged 55 and over, and there were major methodological changes between 2014 and 2017, thus constraining comparisons. Ethical approval was obtained to conduct the IHP by the CWF and no additional steps were required for secondary analysis of IHP survey data.

### Health insurance system in Switzerland

In Switzerland, since the introduction of a major, national, health insurance reform in 1994, healthcare financing relies on compulsory health insurance combined with a cost-sharing model based on annual deductibles and co-insurance. The level of deductibles chosen by individuals varies, and the highest deductibles (CHF 2500) are less financially burdensome than the lowest deductibles (CHF 300) [16]. Coinsurance starts when the annual deductibles are entirely paid and will imply for the individuals to pay 10% of all covered expenses up to a maximum of CHF 700 per year.

### Study population

For this analysis, we used data from participants of the 2017, 2021 and 2024 IHP surveys conducted in Switzerland. The CWF designed the international survey and commissioned the SSRS (Social Science Research Solutions) statistics company to collect and process the data. The Federal Office of Public Health (FOPH) oversaw the project and mandated the Swiss Health Observatory (Obsan) to analyse the data and produce the national report [17]. The Swiss sample was drawn by the Swiss Federal Statistical Office (FSO) from the population to extract a representative sample of the population aged 65 years or older. The targeted population, random sample, eligible sample invited to participate, and analytical sample are presented in Supplementary Table S1. Due to legal confidentiality reasons, the FSO could not provide the exact number of people residing in Switzerland at the time of recruitment in 2017. Accordingly, the number of people residing in Switzerland at the time of recruitment is based on aggregated data from the FSO’s interactive tables [18]. The participation rate in Switzerland was 44.6% in 2017, 47.5% in 2021, and 48.9% in 2024. While the survey aims to obtain a representative sample of people aged 65 and over in Switzerland, the 2017 survey over-sampled French-speaking participants and under-sampled German-speaking participants, and analytical weights were adjusted accordingly (see below in the statistical analysis section).

### Data collection and measurement

The study participants completed the questionnaire by phone or online between March and May 2017, March and June 2021, and between March and June 2024, respectively. Participants provided multiple information, including on age, gender, socioeconomic position, self-reported morbidities and self-rated health [19–21].

Our outcome was forgone medical and dental care due to cost. We assessed forgone care based on participants’ responses to 4 questions regarding forgoing medical prescriptions, consultations, medical tests, treatments or follow-up consultations and dental visits by the participant. We defined «insurance-covered services» as those included in the mandatory basic insurance plan, which encompasses virtually all healthcare services except dental care. Thus, our three indicators of forgone care were: forgone insurance-covered services, dental care, and all care (insurance-covered services and dental care combined).

Other indicators used in our analyses were age, gender, education level, monthly household income, self-rated health and morbidities, and their categories are shown and regrouped in Table 1. We categorised education as primary (International Standard Classification of Education—ISCED—2011 1-2, or pre-primary and primary education), secondary (ISCED 2011 2-3-4, or lower or upper secondary education, or post-secondary non-tertiary education) and tertiary (ISCED 2011 5-6, or first- or second-stage tertiary education). Household income was assessed with categorical variables, and we regrouped it in three categories, i.e. less than CHF 5000 per month, CHF 5000–9000 per month, and CHF 9000 or more. Self-rated health (SRH) was assessed with the following question, ‘In general, how would you define your own health?’ (response categories according to the US SRH categories: excellent, very good, good, fair, poor). Morbidities were self-reported, and participants could report the following morbidities: hypertension or high blood pressure, heart disease (including heart attack), diabetes, asthma or chronic lung disease (such as chronic bronchitis, emphysema or COPD), depression, anxiety or other mental health conditions, cancer, joint pain or arthritis, ‘had a stroke’, and neurological problems (like dementia/Alzheimer’s disease).

**Table 1.** Characteristics of the participants (N^17^ = 2570; N^21^ = 1888; N^24^ = 1948)^a^^,^^b^

Characteristics		2017	2021	2024
Gender	Men	1245 (46)	915 (46)	943 (46)
	Women	1325 (54)	973 (54)	1006 (54)
Age (years)	Mean (SD)	74.6 (6.7)	74.8 (6.6)	74.8 (6.8)
	65–79	1990 (76)	1460 (75)	1467 (74)
	80+	580 (24)	428 (25)	481 (26)
Language	French	1491 (25)	792 (25)	526 (25)
	Italian	257 (7)	208 (6)	255 (6)
	German	822 (68)	888 (70)	1167 (68)
Education level	Primary	557 (24)	401 (23)	342 (17)
	Secondary	1419 (64)	1009 (67)	1182 (71)
	Tertiary	594 (12)	478 (10)	424 (13)
Monthly household income	Less than 5'000 CHF	1251 (54)	937 (52)	900 (47)
	5'000 to 8'999 CHF	876 (34)	664 (38)	760 (38)
	9'000 CHF or more	443 (13)	287 (10)	288 (14)
Self-rated health	Excellent or very good	805 (30)	603 (30)	540 (28)
	Good	1224 (49)	924 (52)	919 (46)
	Fair or poor	536 (21)	358 (18)	466 (25)
Morbidities	Arterial hypertension	1257 (49)	932 (52)	918 (48)
	Cardiac	548 (22)	355 (20)	442 (23)
	Diabetes	371 (14)	246 (12)	274 (14)
	Pulmonary	299 (11)	243 (14)	225 (12)
	Psychiatric	351 (12)	227 (10)	214 (12)
	Cancer	377 (14)	292 (16)	294 (16)
	Arthritis	1172 (43)	754 (40)	765 (41)
	Stroke	171 (6)	108 (5)	126 (6)
	Neurological	60 (2)	41 (2)	66 (3)
	Multimorbidity	1369 (51)	939 (52)	989 (53)

a Results are shown as *N* (weighted percentages).

b Note: CHF = Swiss francs. Education categories reflect the International Standard Classification of Education (ISCED) 2011 version.

### Statistical analysis

For our analysis, we performed a complete case analysis and excluded all data that were missing, not applicable, rated as ‘other’, where the participant was not sure or when they declined to answer. This does not apply for morbidities and self-rated health characteristics, since we did not use them for further analyses. The number of each of these deleted cases is shown in Supplementary Table S2. Our final analytical sample was 6406 participants (2017: 2570; 2021:1888; 2024: 1948) who completed the study across the years.

We first presented descriptive statistics on the characteristics and cost-related forgone medical services of the population aged 65 and over in Switzerland. All percentages were calculated using weighted analyses. Weights were provided by the Commonwealth Fund to account for unequal probabilities of selection due to stratification and oversampling and were calibrated to match the demographic distribution of the Swiss 65+ population by age, sex, and region. We reported weighted analyses in the paper. Except for language distribution in 2017 (and to a lesser extent in 2021), unweighted analyses were conducted and yielded similar results (Supplementary Table S3).

Second, we assessed the disparities in forgone care according to gender, age groups, education level and income groups, using stratification.

Third, we used the index of disparity, defined as ‘the average of the absolute differences between rates for specific groups within a population and the overall population rate, divided by the rate for the overall population and expressed as a percentage’ [14]. It summarizes the average difference between group rates and the total population rate. It is calculated using the following formula:


$$\text{Index}\;\text{of}\;\text{disparity}=\left(\Sigma \;\left|r_{\left(1–n\right)}–\;R\right|/n\right)/R*100$$

where r = group rate and R = total population rate. This index provides a summary measure of disparity across multiple subgroups (e.g. income or education levels) and is similar to the coefficient of variation. It considers the distribution of rates across all groups and expresses disparity relative to the population average, allowing for comparisons across indicators and over time. This index is expressed in percentages and can exceed 100%. An index of disparity equal 0 means that there are no disparities, and it increases with increasing disparities. In our case, the rates were the prevalence of forgone care, and the groups we compared were across education and income strata.

### Writing and editing assistance

Language editing assistance for improving clarity and fluency of the English manuscript was provided by a large language model (ChatGPT^®^ by OpenAI).

## Results

The characteristics of participants, as well as weighted percentages per groups, are shown in Table 1. Their socio-demographic profile remained relatively stable across surveys. The mean age was around 75 years across all survey waves. 54% of participants were women, and about 25% were aged 80 or older. The distribution of languages, education levels, and income groups remained stable across the three survey waves. Approximately 20% of participants had primary education, and nearly half reported a household income below CHF 5000 per month. Self-rated health was consistent over time, with around 30% rating it as excellent or very good, 50% as good, and 20% as fair or poor. Multimorbidity (defined as two or more chronic conditions) affected approximately half of the participants. The most prevalent morbidities were hypertension (about 50%), arthritis (40%), followed by diabetes and cancer (14%–16%).

Table 2 shows the weighted prevalence of cost-related forgone care over time, with the most important results stratified by gender and age. In 2024, the prevalence of older adults having forgone at least one health service due to cost was 20%, matching the 21% reporting it in 2017 after a transient decrease to 16% in 2021. Forgone services covered by basic insurance plans remained stable at 13% in 2017 and 13% 2024, and dropped to 9% in 2021. Dental care was consistently the most frequently forgone service, with 13% in 2017 and 2024, and 11% in 2021. In each survey, men and participants aged 65–79 years tended to report higher levels of forgone care than women and those aged 80 or older, respectively.

**Table 2.** Forgone care due to cost among older adults, Switzerland, in 2017, 2021, and 2024^a^

Forgone care	Subset	2017	2021	2024
*During the past 12 months, was there a time when you…*				
Did not collect a prescription for medicine, or you skipped doses of your medicine because of the cost?^b^	All	4.8% (3.7–6.1)	3.4% (2.4–4.7)	6.0% (4.7–7.6)
Had a medical problem but did not consult with a doctor because of the cost?^b^	All	6.8% (5.5–8.4)	4.8% (3.6–6.4)	7.4% (5.9–9.2)
Skipped a medical test, treatment, or follow-up that was recommended by a doctor because of the cost?^b^	All	5.2% (4.1–6.5)	4.2% (3.2–5.7)	6.8% (5.4–8.7)
Forgoing at least one insurance-covered health service	All	12.5% (10.8–14.5)	8.5% (6.9–10.4)	13.2% (11.3–15.5)
	Men	14.6% (11.9–17.9)	10.5% (8.0–13.8)	14.1% (11.3–17.6)
	Women	10.7% (8.6–13.4)	6.7% (4.9–9.1)	12.5% (9.9–15.6)
	65–79 years	13.8% (11.7–16.2)	9.9% (8.0–12.4)	14.1% (11.9–16.8)
	80 years or above	8.7% (5.9–12.6)	4.1% (2.3–7.3)	10.9% (7.4–15.7)
*During the past 12 months, was there a time when you did not visit a dentist when you needed to because of the cost?*	All	13.0% (11.3–14.9)	10.8% (9.1–12.8)	13.0% (11.1–15.2)
	Men	13.3% (10.9–16.2)	11.7% (9.2–14.8)	13.0% (10.3–16.3)
	Women	12.7% (10.4–15.4)	10.0% (7.8–12.7)	13.0% (10.5–16.0)
	65–79 years	14.2% (12.2–16.5)	11.5% (9.5–13.8)	13.8% (11.6–16.3)
	80 years or above	9.2% (6.4–13.0)	8.7% (5.8–12.9)	11.0% (7.7–15.5)
Forgoing at least one health service	All	20.6% (18.5–22.9)	16.3% (14.1–18.7)	20.1% (17.8–22.7)
	Men	22.7% (19.5–26.3)	19.1% (15.8–23.0)	22.2% (18.7–26.2)
	Women	18.8% (16.0–21.9)	13.9% (11.2–17.0)	18.4% (15.4–21.8)
	65–79 years	22.4% (19.9–25.1)	17.9% (15.3–20.8)	21.5% (18.8–24.5)
	80 years or above	15.1% (11.4–19.7)	11.6% (8.1–16.3)	16.5% (12.4–21.7)

a Results are shown as weighted percentages (95% confidence interval).

b Insurance-covered health services.

**Figure 1.**
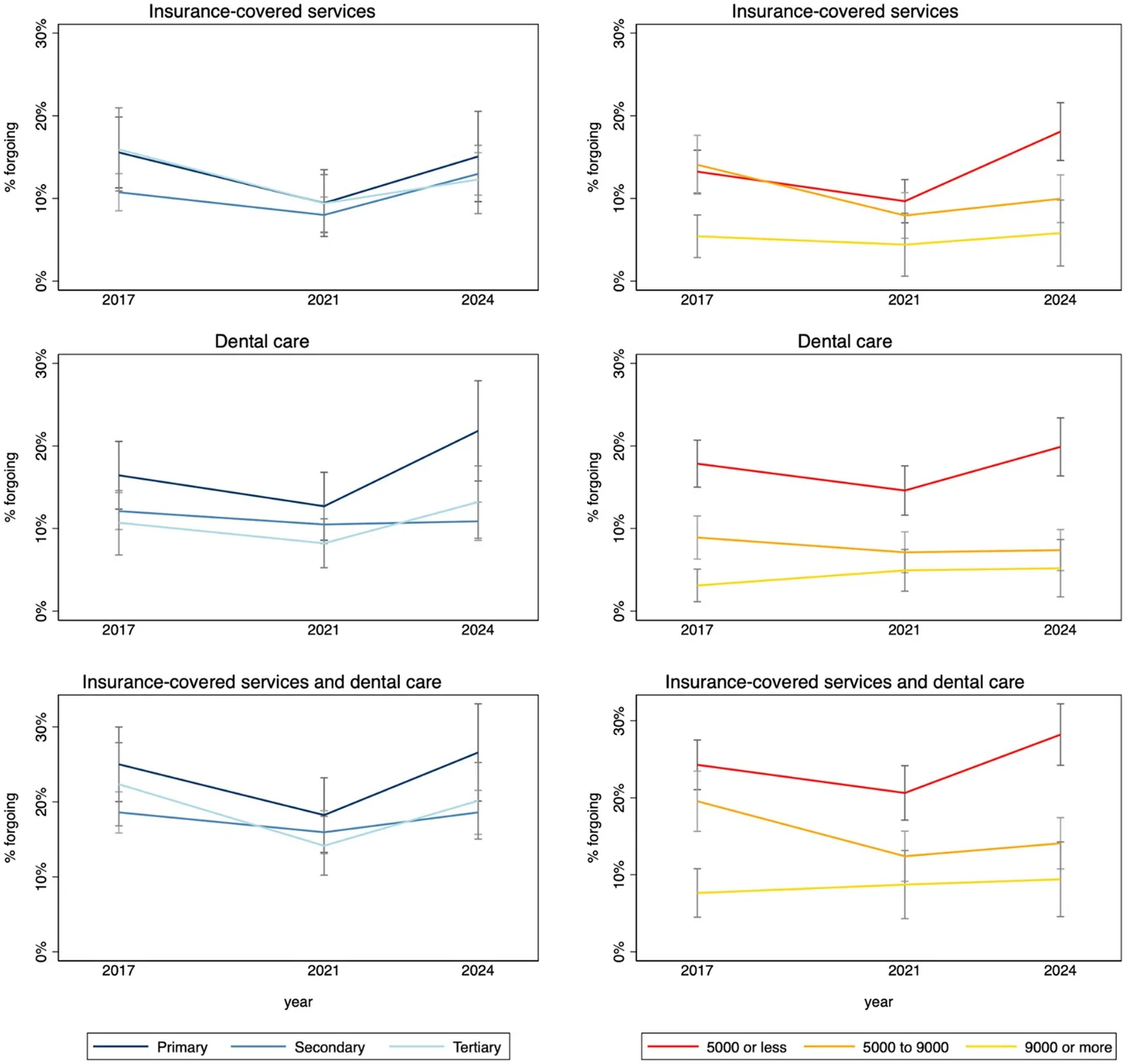
Forgone insurance-covered services, dental care, and insurance-covered services and dental care combined due to cost, by education level and by monthly household income (Swiss francs) among Swiss older adults (2017–2024).

**Figure 2.**
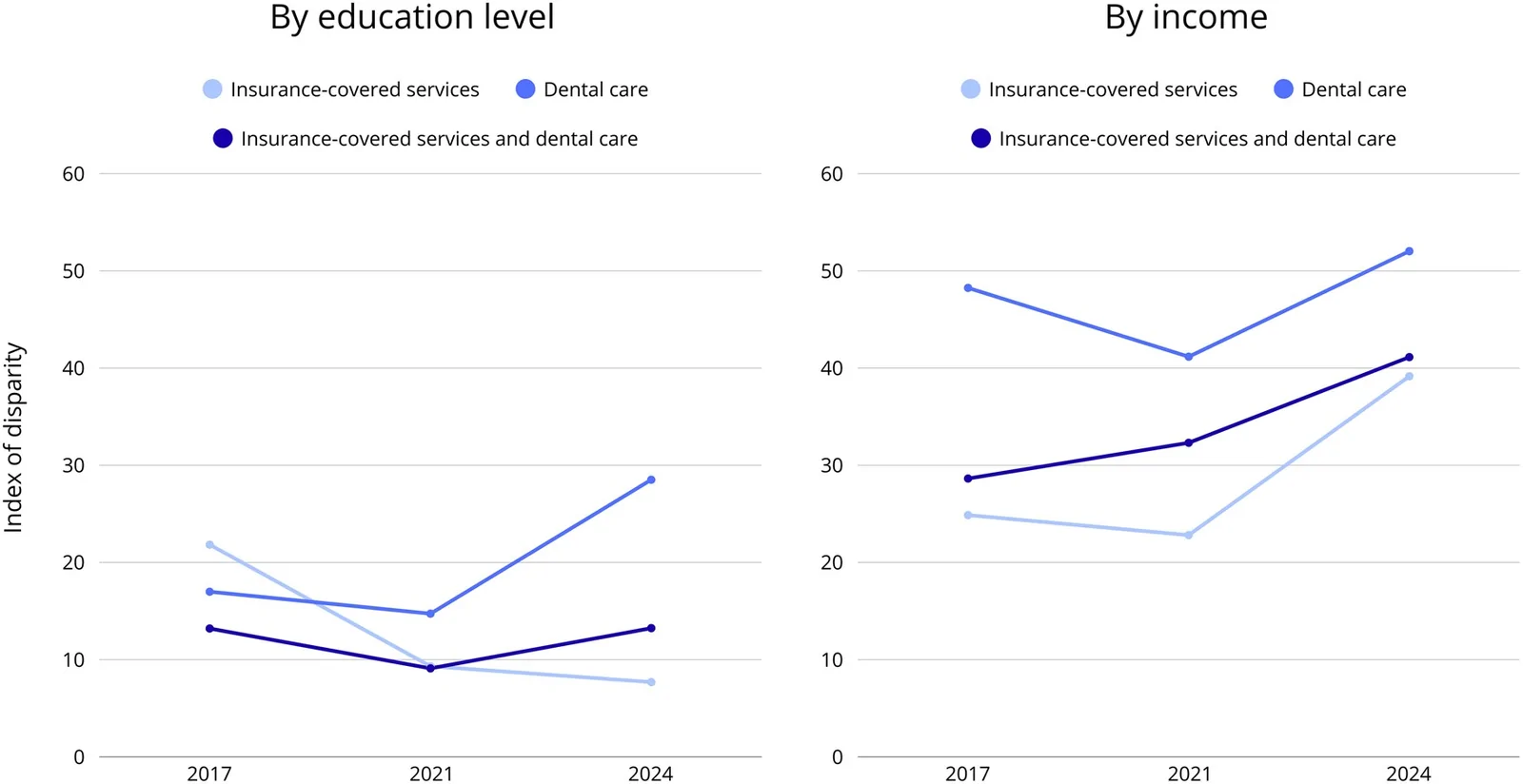
Index of Disparity by education level and by income for cost-related forgone healthcare, among Swiss older adults (2017–2024).

Figure 1 shows the trends in disparities in cost-related forgone care by education and income levels. Disparities in forgone care by income were relatively large and increased over time. Disparities by education level were smaller and remained relatively stable over time. These findings were confirmed by the disparity index (Fig. 2), which showed a steady rise in income-related disparities between 2017 and 2024, with the highest disparities found in dental care. Notably, income-based disparities also increased for services potentially covered by compulsory insurance plans. The disparity index based on education showed only minimal variation across years, except for dental care. Across all three indicators of forgone care (i.e. forgone insurance-covered services, dental care, and all care combined), income-based disparities were consistently greater than those based on education. For instance, in 2024, the disparity by income in the forgoing of all care was 41%, compared to 13% for education.

## Discussion

In this study, we investigated trends in cost-related forgone care among adults aged 65 years and older in Switzerland, with a focus on socioeconomic disparities. In 2024, one in five Swiss adults aged 65 years or older reported forgoing care due to cost. Forgone care affected all types of services, including prescribed medications, medical consultations, and recommended tests or treatments. Women and adults aged 80 years or older reported lower rates of forgone care compared to men and adults aged 65–79 years, respectively. Despite Switzerland’s compulsory health insurance system, aiming to ensure equal healthcare provision, our results highlight persistent and widening disparities by income. The proportion of forgone care was similar in 2017 and 2024, with a transient decrease in the 2021 survey that took place at the time of the COVID-19 pandemic. There were high and increasing income-related disparities in forgone care, whereas disparities according to education level were lower and remained relatively stable over time. The highest disparities were observed for dental care, a service not covered by basic health insurance in Switzerland. Our results suggest that disparities related to healthcare provision are influenced more by material factors, such as income, than by social or cultural factors, such as education.

Comparisons across studies are challenging because definitions of forgone care can vary, and the terms ‘forgone care’ and ‘unmet healthcare need’ are often used interchangeably, although not strictly equivalent. Unmet healthcare need refers to a broader concept encompassing healthcare access more generally [22], which cost-related forgone care does not fully capture. In our study, several survey items refer to situations where individuals had formal access to healthcare but did not obtain recommended services because of cost. Moreover, the clinical value of forgone care is unknown, limiting conclusions about whether the care is necessary.

Our findings align with international evidence that even in high-income countries, income disparities are associated with unequal access to healthcare: across OECD countries, people in the lowest income quintile were around 2.5 times more likely to report unmet medical care needs than those in the highest income quintile [23]. Like our study, data from the 2024 European EU-SILC study revealed that the share of unmet needs for dental care exceeded unmet needs for medical care [24]. This study also reveals that rates of unmet needs in Switzerland are close to the middle of the distribution score in Europe, despite the country’s prosperity. In France, about 6% of adults situated in the lowest income quintile reported unmet medical needs due to cost, distance to travel or waiting times, compared with 1% among the highest quintile [25], and in Sweden, 1.7% versus 0.6% respectively [26]. Interestingly, the decrease in healthcare renunciation in 2021 during the COVID-19 pandemic was also observed among older adults in Italy [27]. In Switzerland, the level of deductible chosen by insured individuals and the coinsurance may further amplify disparities, as those with lower incomes are more likely to delay or avoid care to reduce costs, potentially exacerbating health problems over time. In Geneva, one population-based study conducted in 2008/9 among adults aged 35–74 years showed that 15% renounced health care for economic reasons, and this percentage decreased with higher income [28]. While the Swiss Federal Office of Public Health already described cost-related forgone care from 2017 to 2024 [17], our findings go further by quantifying the growing disparities underlying this trend. The use of the index of disparity provides a tool to quantify these disparities, revealing widening differences between income groups over the past decade.

This study has several limitations. First, as a secondary analysis of survey data, our study was strongly constrained by the surveys. As such, although it would have been interesting to describe forgone care by wealth groups (i.e. bank account holdings), the IHP survey did not include this information, and we were therefore unable to examine it. Second, participation was voluntary, which may have introduced a healthy volunteer bias, thus limiting the generalizability of our findings. As all data were self-reported, we cannot exclude recall bias, to which older adults are more prone [29]. Social desirability in self-reported measures may lead to underreporting cost-related forgone care. Furthermore, individuals were reached by phone or online, which may have resulted in underrepresentation of participants with lower socioeconomic position. Third, the cross-sectional design of the surveys and the statistical methods do not allow for causal inference. Fourth, one major limitation is that we do not have any information on the value of forgone care, as it would be less problematic if low-value rather than high-value care were forgone [30]. Fifth, the 2021 survey took place during COVID-19, thus limiting comparability across surveys. Lastly, cost-related forgone care is not a direct measure of healthcare access, which is broader and depends on the presence of healthcare need as well as non-financial barriers. Because the International Health Policy Survey does not include a direct measure of healthcare needs, we could not construct need-based estimates, such as the share of the population with unmet need (number of people with unmet need divided by number reporting a need), as used in recent Eurostat indicators [31]. Our findings may therefore reflect trends in financial barriers to care rather than access per se.

In conclusion, in a context of high cost-sharing within the health insurance system and despite compulsory health insurance, one in five older adults in Switzerland still forgo care for financial reasons, and income disparities have widened. In the context of an ageing population, these trends demand policy attention to help strengthen monitoring of Switzerland’s health coverage [32]. Efforts should focus on strengthening equity in health financing. Ensuring that all individuals–regardless of income–can access necessary healthcare without financial hardship is essential for a fair and sustainable health care system.

## Supplementary Material

ckag010_Supplementary_Data

## Data Availability

Data are publicly available by contacting the CWF or local health agencies of the participating countries. The code used to perform the analyses of this study can be shared upon request.
