# Supplementary material for: Trends in cost-related forgone care among older adults in Switzerland: a repeated cross-sectional study
Source: Eur J Public Health. 2026 Feb 11;36(2):ckag010. doi: 10.1093/eurpub/ckag010 (PMC13017530; doi:10.1093/eurpub/ckag010)
Supplement: ckag010_Supplementary_Data [file ckag010_supplementary_data.docx]

**Trends in cost-related forgone care among older adults in Switzerland: a repeated cross-sectional study**

**Dr. Mathieu Jendly^1^, PD Dr. Stéphane Cullati^1,2^, Dr. Cornelia Wagner^1,3^, Dr. Axelle Braggion^1,3^, Prof. Valérie Santschi^4^, Prof. Arnaud Chiolero^1,2,5,6*^**

^1^ Population health laboratory (#PopHealthLab), University of Fribourg, Fribourg, Switzerland

^2^ Swiss Centre of Expertise in Life Course Research, Geneva & Lausanne, Switzerland

^3^ Swiss School of Public Health (SSPH+), Zurich, Switzerland

^4^ La Source, School of Nursing Sciences, HES-SO University of Applied Sciences and Arts Western Switzerland, Lausanne, Switzerland

^5^ School of Population and Global Health, McGill University, Montreal, QC, Canada

^6^ Institute of Primary Health Care (BIHAM), University of Bern, Bern, Switzerland

*** Correspondence:**Dr. Mathieu Jendly
Population health laboratory
University of Fribourg
Route des Arsenaux 41
1700 Fribourg
mathieu.jendly@unifr.ch

**Appendix**

**Supplementary Table S1**: Target population, random sample, individuals invited to participate, participants, and participation rate, in 2017, 2021, and 2024.

| Year | Target population^1^ | Random sample^2^ | Ineligibles^3^ | Population invited to participate^4^ | Participants^5^ | Participation rate^6^ | Analytical sample^7^ |
| --- | --- | --- | --- | --- | --- | --- | --- |
| 2017 | 1550365 | 7424 | 163 | 7261 | 3238 | 44.6% | 2570 |
| 2021 | 1555231 | 5505 | 33 | 5472 | 2597 | 47.5% | 1888 |
| 2024 | 1645002 | 5633 | 230 | 5403 | 2634 | 48.8% | 1948 |

1: individuals aged 65 and living in Switzerland at the start of the survey.

2: random sample from the study population provided by the Swiss Federal Statistical Office.

3: participants excluded from the random sample (no answer or busy, call-blocking, non-working number, no eligible respondent).

4: valid sample after exclusion ineligibles.

5: sample who completed the questionnaire.

6: participants divided by the valid sample.

7: participants selected for the complete case analysis.

**Supplementary Table S2**: Number of missing and alternative cases for each variable deleted for the complete case analysis, in 2017, 2021, and 2024. Some data were not available (NA).

| Year | Variable | Missing | Not applicable | Other | Not sure / Uncertain | Declined to answer |
| --- | --- | --- | --- | --- | --- | --- |
| 2017 | Gender | 0 (0) | *NA* | *NA* | *NA* | 0 (0) |
|  | Age | 0 (0) | *NA* | *NA* | *NA* | 0(0) |
|  | Language | 0 (0) | *NA* | *NA* | *NA* | *NA* |
|  | Education level | 0 (0) | *NA* | *NA* | 14 (0) | 13 (0) |
|  | Income | 0 (0) | *NA* | *NA* | 107 (3) | 153 (5) |
|  |  |  |  |  |  |  |
|  |  | |  |  |  |  |
|  | *Forgone…* | |  |  |  |  |
|  | Medicine | 0 (0) | 195 (6) | *NA* | 0 (0) | 0 (0) |
|  | Consultation | 0 (0) | 197 (6) | *NA* | 1 (0) | 0 (0) |
|  | Follow-up | 0 (0) | 235 (7) | *NA* | 0 (0) | 0 (0) |
|  | Dentist | 0 (0) | 190 (6) | *NA* | 0 (0) | 0 (0) |
| 2021 | Gender | 0 (0) | *NA* | *NA* | *NA* | 2 (0) |
|  | Age | 0 (0) | *NA* | *NA* | *NA* | 0(0) |
|  | Language | 0 (0) | *NA* | *NA* | *NA* | *NA* |
|  | Education level | 0 (0) | *NA* | *NA* | 165 (6) | 104 (4) |
|  | Income | 0 (0) | *NA* | *NA* | 23 (1) | 149 (6) |
|  |  |  |  |  |  |  |
|  |  | |  |  |  |  |
|  | *Forgone…* | |  |  |  |  |
|  | Medicine | 0 (0) | 193 (7) | *NA* | 0 (0) | 3 (0) |
|  | Consultation | 0 (0) | 178 (7) | *NA* | 0 (0) | 3 (0) |
|  | Follow-up | 0 (0) | 186 (7) | *NA* | 0 (0) | 4 (0) |
|  | Dentist | 0 (0) | 164 (6) | *NA* | 0 (0) | 4 (0) |
| 2024 | Gender | 0 (0) | *NA* | 12 (0) | *NA* | 46 (2) |
|  | Age | 0 (0) | *NA* | *NA* | *NA* | 7 (0) |
|  | Language | 0 (0) | *NA* | *NA* | *NA* | *NA* |
|  | Education level | 0 (0) | *NA* | *NA* | 2 (0) | 14 (1) |
|  | Income | 0 (0) | *NA* | *NA* | 31 (1) | 81 (3) |
|  |  |  |  |  |  |  |
|  |  | |  |  |  |  |
|  | *Forgone…* | |  |  |  |  |
|  | Medicine | 0 (0) | 296 (11) | *NA* | 0 (0) | 44 (2) |
|  | Consultation | 0 (0) | 281 (11) | *NA* | 0 (0) | 52 (2) |
|  | Follow-up | 0 (0) | 282 (11) | *NA* | 0 (0) | 51 (2) |
|  | Dentist | 0 (0) | 278 (11) | *NA* | 0 (0) | 43 (2) |

**Supplementary Table S3**: Characteristics of the participants (N^17^ = 2570; N^21^ = 1888; N^24^ = 1948). Results are shown as N (unweighted percentages).

| Characteristics |  | 2017 | 2021 | 2024 |
| --- | --- | --- | --- | --- |
| Gender | Men | 1245 (48) | 915 (48) | 943 (48) |
|  | Women | 1325 (52) | 973 (52) | 1006 (52) |
| Age (years) | Mean (SD) | 74.6 (6.7) | 74.8 (6.6) | 74.8 (6.8) |
|  | 65-79 | 1990 (77) | 1460 (77) | 1467 (75) |
|  | 80+ | 580 (23) | 428 (23) | 481 (25) |
| Language | French | 1491 (58) | 792 (42) | 526 (27) |
|  | Italian | 257 (10) | 208 (11) | 255 (13) |
|  | German | 822 (32) | 888 (47) | 1167 (60) |
| Education level | Primary | 557 (22) | 401 (21) | 342 (18) |
|  | Secondary | 1419 (55) | 1009 (53) | 1182 (61) |
|  | Tertiary | 594 (23) | 478 (25) | 424 (22) |
| Monthly houshold income | Less than 5'000 | 1251 (49) | 937 (50) | 900 (46) |
|  | 5'000 to 8'999 | 876 (34) | 664 (35) | 760 (39) |
|  | 9'000 or more | 443 (17) | 287 (15) | 288 (15) |
| Self-rated health | Excellent or very good | 805 (31) | 603 (32) | 540 (28) |
|  | Good | 1224 (48) | 924 (49) | 919 (47) |
|  | Fair or poor | 536 (21) | 358 (19) | 466 (24) |
| Morbidities | Arterial hypertension | 1257 (49) | 932 (49) | 918 (47) |
|  | Cardiac | 548 (21) | 355 (19) | 442 (23) |
|  | Diabetes | 371 (14) | 246 (13) | 274 (14) |
|  | Pulmonary | 299 (12) | 243 (13) | 225 (12) |
|  | Psychiatric | 351 (14) | 227 (12) | 214 (11) |
|  | Cancer | 377 (15) | 292 (15) | 294 (15) |
|  | Arthritis | 1172 (46) | 754 (40) | 765 (39) |
|  | Stroke | 171 (7) | 108 (6) | 126 (6) |
|  | Neurological | 60 (2) | 41 (2) | 66 (3) |
|  | Multimorbidity | 1369 (47) | 939 (50) | 989 (49) |

Note : CHF= Swiss francs. Education categories reflect the International Standard Classification of Education (ISCED) 2011 version.
